# Volumetric and Intrastructural Characterization of the Substantia Nigra Using Optimized Synthetic MR Images Derived From Quantitative T1 Maps at 3 T

**DOI:** 10.1002/nbm.70421

**Published:** 2026-10-08

**Authors:** Lukas von Erdmannsdorff, Lora Kovacheva, Dennis C. Thomas, Elke Hattingen, Ralf Deichmann, Christophe Théo Arendt

**Affiliations:** ^1^ Goethe University Frankfurt University Hospital, Institute of Neuroradiology Frankfurt am Main Germany; ^2^ Goethe University Frankfurt Cooperative Brain Imaging Center – CoBIC Frankfurt am Main Germany

**Keywords:** aging, brain mapping, magnetic resonance imaging, Parkinson's disease, substantia nigra

## Abstract

Accurate imaging of the substantia nigra (SN) is critical for diagnosing and monitoring neurodegenerative conditions like Parkinson's disease. However, conventional magnetic resonance imaging (MRI) struggles to precisely visualize this small structure within the deep brain due to poor intrinsic tissue contrast. Although quantitative MRI (qMRI) captures objective tissue properties with diagnostic value to assess neurodegenerative conditions of the SN, additionally acquiring multiple MRI contrasts inherently requires impractically long scan times. To overcome this, qMRI‐derived synthetic MRI uses mathematical signal models to retrospectively generate various synthetic contrast weightings from a single map of quantitative T1‐relaxation times (qT1). This study investigated which mathematical transformations of qT1 maps, including an approximation for pseudo‐proton spin density (pPSD), are best suited to derive optimized synthetic MRI contrasts for accurate SN analysis. By evaluating six dedicated qT1‐derived contrast weightings in healthy subjects, this work demonstrates that a nonlinear, signal‐increased pPSD‐based synthetic MRI contrast (si^4^_PSD) yields a favorable balance by significantly enhancing optical contrast while maintaining a sufficient signal‐to‐noise ratio and preserving contrast‐to‐noise ratio. The si^4^_PSD images enabled highly consistent, automated atlas‐based segmentation of the SN that significantly outperformed traditional manual delineation, overcoming rater variability and bias from age‐dependent contrast changes. Automated segmentation revealed significant sex‐ and hemisphere‐dependent differences in SN volume, whereas qT1 data simultaneously uncovered distinct subregional and asymmetric intrastructural aging patterns. These findings highlight that macrostructural volume and intrastructural tissue properties must be assessed as independent variables. In conclusion, this qT1‐based, automated framework offers a highly consistent, scalable approach to SN mapping without extending patient scan times. By clearly separating physiological intrastructural changes affecting MRI contrasts from mistakenly perceived general atrophy, this methodology provides a powerful foundation for developing more precise and objective imaging biomarkers for SN‐related neurodegenerative diseases in both research and clinical settings.

## Graphical abstract

Automated segmentation using the NextBrain atlas and synthetic MRI effectively overcomes systematic age‐related biases observed in manual delineation of the substantia nigra. A novel qT1‐based synthetic contrast utilizing signal‐increased pseudo‐proton spin density (pPSD) provides improved intrastructural separation of nigral subregions (SNpc and SNpr) for consistent automated quantification in future studies. By decoupling volumetric estimates from intrastructural tissue properties (qT1), this scalable framework reveals distinct and potentially asymmetric aging patterns in SN subregions.

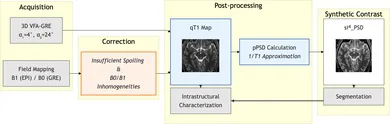

**Abbreviations**
CI: confidence interval
CNR: contrast‐to‐noise ratio
CSF: cerebrospinal fluid
ci_T1: contrast‐increased T1 map
GRE: gradient echo
ICC: intraclass correlation coefficient
ICV: intracranial volume
IQR: interquartile range
IR_WM: synthetic inversion recovery with white matter nulling
MP‐RAGE: magnetization‐prepared rapid gradient echo
MRI: magnetic resonance imaging
MT: magnetization transfer
NM: neuromelanin
NM‐MRI: neuromelanin‐sensitive MRI
OC: optical contrast
PD: Parkinson's disease
pPSD: pseudo‐proton spin density
PSD: proton spin density/synthetic PSD‐weighted images
qMRI: quantitative MRI
qT1: quantitative T1
si^4^_PSD: synthetic signal‐increased PSD‐weighted images
SN: substantia nigra
SNpc: substantia nigra pars compacta
SNpr: substantia nigra pars reticulata
SNR: signal‐to‐noise ratio
T1_MP: synthetic T1‐weighted MP‐RAGE
T1_PSD_MP: synthetic T1‐weighted MP‐RAGE with pPSD signal influence

## 1 Introduction

The selective loss of dopaminergic neurons in the pars compacta of the substantia nigra (SN) is a hallmark of Parkinson's disease (PD) [1]. Although MRI is not required for clinical diagnosis or routine monitoring of PD and is primarily used to exclude alternative or atypical causes, the development of reliable imaging biomarkers could support diagnosis and disease stratification. However, precise in vivo imaging of the SN remains challenging for two major reasons: Firstly, the structure's small size and poor contrast‐to‐noise ratios (CNR) in conventional MRI, limiting not only its visualization but also accurate segmentation and volumetric assessment [2, 3]; secondly, although neuromelanin‐sensitive MRI (NM‐MRI) addresses those contrast limitations, it suffers from a lack of standardized protocols, which hinder data comparability across different centers and patients [4].

We aimed to address these challenges by using quantitative MR imaging (qMRI), specifically qT1 mapping, enabling robust derivation of objective tissue parameters (T1‐relaxation times). qT1 has been shown to be sensitive to discriminate patients with PD from healthy controls at an early stage [5, 6]. Our approach builds on recent findings suggesting that the high contrast observed in NM‐MRI may stem from differences in water content and macromolecular composition of dopaminergic neurons rather than solely neuromelanin–iron interactions [7, 8]. Notably, proton spin density (PSD)‐weighted imaging has previously been shown to improve visualization of the SN compared with conventional T2‐weighted imaging, although limited structural delineation has restricted its broader application for reliable segmentation and quantitative analysis [9].

qMRI allows the retrospective generation of various synthetic image contrasts from quantitative tissue parameter maps. Although strictly limited to the underlying acquired protocol, for instance, calculating different T1‐dependent contrast weightings from a single qT1 map, this approach enables postacquisition optimization of derived synthetic image contrasts without additional scan time [10, 11]. Recent developments in qMRI and NM‐MRI, such as STrategically Acquired Gradient Echo (STAGE) and Proton‐density Enhanced Neuromelanin Contrast in Low flip angle‐gradient echo (PENCIL), have demonstrated that PSD‐weighted imaging can selectively enhance the signals of neuromelanin‐containing structures like the SN [12, 13, 14].

The variable flip angle (VFA) technique based on two high‐resolution gradient echo (GRE) acquisitions at different excitation angles enables the measurement of qT1 with subsequent calculation of synthetic contrasts [11]. However, it is important to acknowledge the limitations of T1 modeling methods, as quantitative techniques based on single aqueous pool modeling are prone to bias due to a strong interplay between T1 relaxation and magnetization transfer (MT) mechanisms [15]. As a result, the VFA method yields an effective T1 and an effective PSD, the latter still containing residual B_1_‐bias and T2* weighting. Nevertheless, despite their dependence on the specific T1 measurement method, such effective parameters can still be used to generate highly useful synthetic contrasts. Therefore, our approach relies on jointly estimating the effective longitudinal relaxation rate (R1 = 1/T1) and pseudo‐proton spin density (pPSD)‐related parameters, while reducing receive coil intensity bias [16, 17, 18]. Fully quantitative PSD mapping would require additional acquisitions and corrections for B_1_ inhomogeneity and T2* effects [19]. In the present work, we use pPSD solely as a robust transformation of qT1 for synthetic contrast generation [18, 20], an approach that has been shown to significantly improve cerebral tissue segmentation [21]. In this sense, pPSD provides a practical and time‐efficient PSD approximation for qT1‐derived contrast synthesis [18, 19, 20, 21].

Based on this framework, we examined which qT1‐based transformations produce synthetic contrasts that best improve structural delineation of the SN. We combined this analysis with a recently introduced histology‐based probabilistic atlas (NextBrain), which enables robust, automated, and contrast‐independent segmentation of the SN and its subregions for joint assessment of volume and qT1 [22]. The aims of this study were to (i) assess different synthetic MRI contrasts purely derived from qT1 mapping with respect to their suitability for SN segmentation using image quality metrics, and manual and automated methods; (ii) investigate demographic and subregional differences in volumes within the total SN, pars compacta (SNpc), and pars reticulata (SNpr); and (iii) evaluate demographic and subregional differences in qT1 values across the same regions.

This initial analysis in healthy controls served to evaluate and optimize qT1‐derived synthetic contrasts for substantia nigra delineation and provides the methodological foundation for what must be accounted for when establishing reference values in future clinical studies, particularly in patients with PD.

## 2 Materials and Methods

### 2.1 Study Population

This retrospective study analyzed data from 18 adult healthy volunteers (nine women [median age 44 years, IQR 33–53] and nine men [median age 45 years, IQR 33–54]; overall median age 44.5 years, range 22–70 years). Participants were recruited as a control group within a prospective, monocentric observational study approved by the local ethics committee (reference number 20–838) [23]. Exclusion criteria included neurological conditions such as PD and other parkinsonian disorders, known neuroinflammatory or systemic inflammatory conditions, intake of neuroactive and chronic immunosuppressive medication, pregnancy, and standard MRI contraindications. All subjects provided written broad consent for secondary analysis of pseudonymized data.

### 2.2 Data Acquisition and Processing

MRI was performed on a 3 T scanner (MAGNETOM Prisma, Siemens Healthineers, Erlangen, Germany) using a 20‐channel phased‐array head coil. Mapping of qT1 and pPSD was performed using two 3‐dimensional VFA‐GRE sequences with FLASH‐EPI hybrid readout (flip angles *α₁* = 4° and *α*_*2*_ = 24°; TR = 16.4 ms; TE = 6.7 ms; 1 mm^3^ isotropic voxels) [24]. B_0_ and B_1_ field correction maps were acquired for correction of field inhomogeneities. Total acquisition time was approximately 12.5 min. Parameter maps were calculated using in‐house MATLAB scripts, including corrections for insufficient spoiling of residual transverse magnetization, as well as B_0_ and B_1_ field inhomogeneities [21, 24, 25, 26]. The acquisition protocol is provided in Table 1.

**TABLE 1:** Acquisition protocol.

Methods	FOV [mm^3^]	TR/TE₁/TE_2_ [ms]	Flip angle α₁/α_2_ [°]	BW [Hz/Pixel]	Voxel size [mm^3^]	Acquisition time [min:s]
3D VFA GRE for qT1 and pPSD mapping	256 × 224 × 160	16.4/6.7	4/24	222	1 × 1 × 1	4:54 and 4:54, i.e., total 9:48
2D GRE for B1 mapping	256 × 224 × 160	11/5	11	260	4 × 4 × 4	1:45
2D dual‐echo GRE for B0 mapping	256 × 224 × 160	560/4.89/7.35	60	200	4 × 4 × 4	1:03

*Note:* The VFA approach involves the acquisition of two 3D gradient echo (GRE) sequences that differ solely in their flip angles, enabling the quantification of T1 relaxation times through the resulting contrast differences. Based on these qT1 values, the proton spin density (PSD) can be approximated using pseudo‐PSD. The reconstructed qT1 maps and the generated synthetic datasets retain the geometrical image properties of the underlying 3D VFA GRE source images.

Abbreviations: 2D = two‐dimensional; 3D = three‐dimensional; B0 = static magnetic field; B1 = radiofrequency excitation field; BW = bandwidth; FOV = field of view; pPSD = pseudo‐proton spin density; qT1 = quantitative T1 relaxation time; TE = echo time; TR = repetition time; VFA = variable flip angle.

Based on these maps, six synthetic image contrast weightings were generated (Figure 1). It is important to note that the following acronyms denote the generated synthetic contrast weightings, not actual quantitative parameter maps:

1. **T1_MP:** Synthetic T1‐weighted magnetization‐prepared rapid gradient echo (MP‐RAGE)‐like images without residual pPSD signal influence [11].
2. **T1_PSD_MP:** Synthetic T1‐weighted MP‐RAGE‐like images with residual pPSD signal influence [11].
3. **IR_WM:** Synthetic inversion‐recovery images with white matter suppression [11].
4. **PSD:** Synthetic PSD‐weighted images (linearly normalized to a fixed white matter reference signal to match standard PSD‐weighted contrast).
5. **si** ^**4**^ **_PSD:** Synthetic nonlinear signal‐increased PSD‐weighted images (pPSD values raised to the fourth power and normalized to the corresponding white matter reference signal) to maximize hyperintense signal separation while preserving natural signal hierarchy.
6. **ci_T1**: Images representing the qT1 map, multiplied with an amplification map for selective contrast enhancement within the range of T1 values between the SN and adjacent white matter (ad). In summary, the amplification map was derived by applying a high‐pass Fermi filter to T1 values, ranging from 1 (low T1) to 2 (long T1), with a value of 1.5 for $0.5\times \left(T1_{\mathrm{AD}}+T1_{\mathrm{SN}}\right)$ and a transition from 1.05 at $T1_{\mathrm{AD}}$ to 1.95 at $T1_{\mathrm{SN}}$.

**FIGURE 1:**
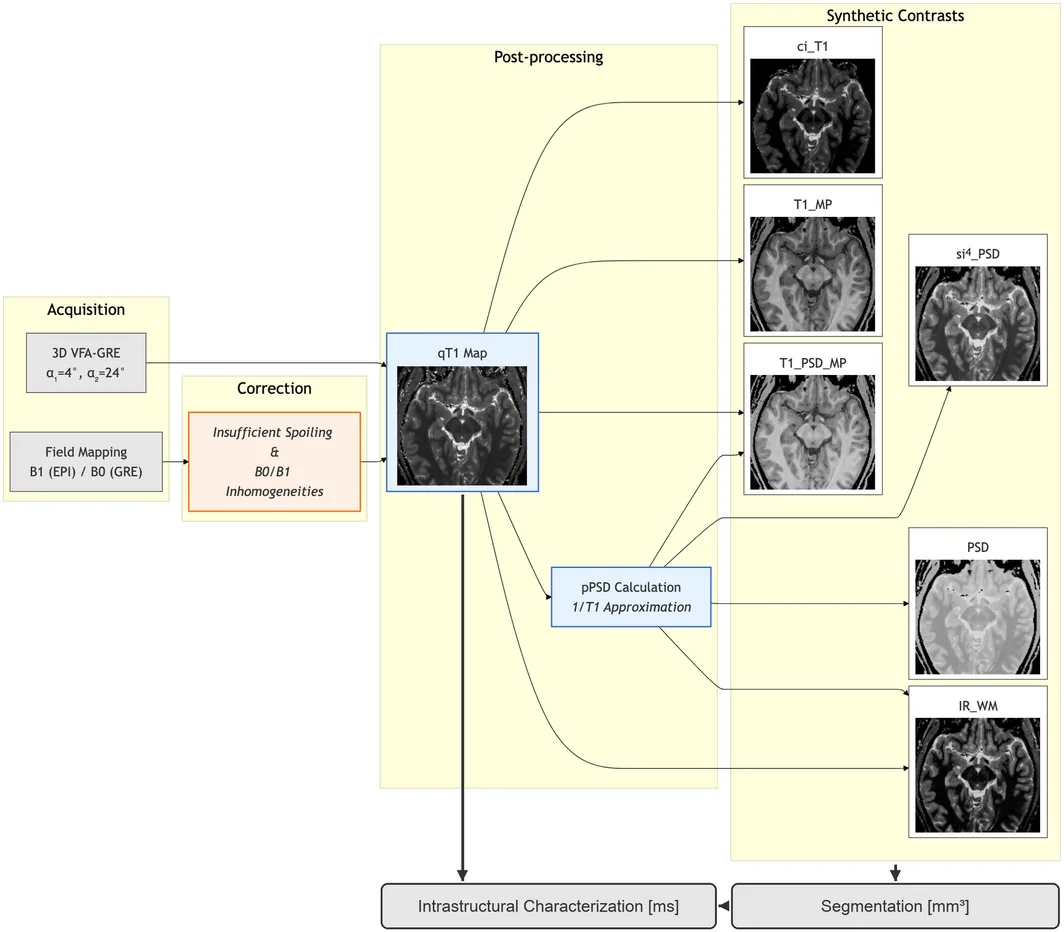
Acquisition and postprocessing workflow for synthetic contrast generation. The pipeline illustrates the processing steps starting from the three‐dimensional VFA‐GRE acquisition. Following correction of insufficient spoiling and B_0_ and B_1_ inhomogeneities, a qT1 map is reconstructed, from which a pseudo‐PSD (pPSD) calculation is mathematically approximated. These quantitative maps serve as a basis for generating six distinct qT1‐derived synthetic image contrast weightings (ci_T1, T1_MP, T1_PSD_MP, si^4^_PSD, PSD, and IR_WM) to evaluate the delineation and segmentation of the substantia nigra with subsequent intrastructural qT1 analysis. Please note that acronyms such as T1_PSD_MP or PSD in this context refer to the respective modeled synthetic contrast weightings, not to quantitative parameter maps. B0 = static magnetic field; B1 = radiofrequency magnetic field; ci = contrast increased; IR_WM = inversion‐recovery–based white matter (nulling); MP = MP‐RAGE‐like; MP‐RAGE = magnetization‐prepared rapid gradient echo; PSD = proton spin density; qT1 = quantitative T1; si^4^ = signal‐increased values raised to the fourth power; VFA‐GRE = variable flip angle‐gradient echo.

For synthetic Datasets 1–5, a global high‐pass Fermi filter was applied postgeneration to suppress background regions with prohibitively low signal‐to‐noise ratios (SNR). To construct this filter, the VFA low‐angle dataset was corrected for T1 weighting and signal nonuniformities and then scaled to an average of 70% across a white matter mask to approximate physiological water content [27]. Although brain tissue PSD typically exceeds 60%, the filter (ranging from 0 to 1) was configured with a cutoff value of 0.5 at 40% and a transition phase between 33% and 47%. Multiplying Datasets 1–5 by this filter maintained full intensities within brain tissue and CSF while effectively masking low‐SNR background areas prone to erroneous T1 fitting. This background‐suppression step is distinct from the T1‐dependent contrast filter used exclusively for Dataset 6 (ci_T1).

In addition, for Datasets 1–5, we specifically utilized the qT1‐derived pPSD rather than the directly available PSD intensity maps obtained via T1‐correction of the low FA image with residual receive coil profile and T2* weighting (PSD*). Because our pipeline exponentiates values to the fourth power to generate the si^4^_PSD contrast, utilizing PSD* would exponentially amplify uncorrected residual receive coil biases in the deep brain. In contrast, deriving pPSD directly from the inherently receive‐bias‐free qT1 map provides a stable foundation for nonlinear contrast generation and subsequent noise estimation, while simultaneously preventing coregistration errors during downstream qT1 value extraction. Thus, for the sole purpose of optimized synthetic contrast generation, utilizing pPSD as an approximation is considered sufficient in the context of this study [21].

Detailed technical specifications for the derived synthetic contrast are provided in Data S1. This qT1 mapping approach has been shown to be sensitive to detect early differences in qT1 values in patients with PD by Baudrexel et al. [5].

#### 2.2.1 Evaluation of Synthetic MRI Contrasts

To evaluate the suitability of synthetic contrasts, both manual and automated segmentation approaches were used, and image quality was assessed using objective and subjective metrics.

##### 2.2.1.1 Manual and Automated Segmentation

Manual SN segmentation was performed by two independent, trained, and blinded raters using MRIcron (version 1.0.2). To assess intercontrast reliability, a single representative midaxial slice of the SN was selected by the lead investigator (verified by a neuroradiologist) for all generated contrasts and presented to the raters in a randomized, blinded order for manual segmentation.

For volumetric analysis, full three‐dimensional manual segmentation of the SN was performed on the contrast that provided the best reliability in ICC and Bland–Altman analyses to ensure the most consistent structural delineation across subjects. Automated segmentation was performed using FreeSurfer 8.0 with the probabilistic NextBrain atlas [22, 28], yielding masks for the total SN, SNpc, and SNpr in subject space. Furthermore, to assess computational reproducibility and allow comparison with manual intrarater reliability, automated segmentation of the same input contrast with the most consistent results was repeated for all subjects.

##### 2.2.1.2 Image Quality Metrics

For the specific calculation of intensity‐based image quality metrics (SNR, CNR, and optical contrast [11]), the masks generated by automated segmentation were eroded (SN: 3 × 3 × 3 voxel kernel; AD: 5 × 5 × 5 voxel kernel) to strictly minimize partial volume effects at the borders. CNR and optical contrast (OC; defined as the relative signal difference between two regions normalized to their mean signal [29], mathematically corresponding to the Michelson contrast) were calculated for the visibility of the SN relative to the adjacent white matter (AD) within the crus cerebri of the midbrain. Noise for SNR and CNR was estimated using analytical derivation combined and compared with Monte Carlo simulation (Data S1). Subjective image quality including SN delineation was rated using a Likert scale (Table S1, Data S2), with a score ≥ 3 considered acceptable. Averaged Likert ratings of both raters were used for further analysis.

#### 2.2.2 Volumetric Analysis

Absolute volumes (in mm^3^) were estimated from the manual segmentations performed by both raters for the total SN and the full, noneroded masks (total SN, SNpc, and SNpr) generated by automated segmentation to capture the complete anatomical extent. The manually segmented midaxial SN‐slices served exclusively to compare the reliability of segmentation between contrasts; consequently, no further analysis was performed on this dataset. Prior to demographic analysis, volumetric results between manual and automated segmentation were compared, and the consistency of automatically segmented volumes was evaluated across the different synthetic contrasts. For the statistical evaluation of volumetric changes regarding age and sex, first, the total SN was assessed, followed by a specific analysis of the subregions.

#### 2.2.3 Intrastructural qT1 Analysis

For the analysis of intrastructural properties, mean qT1 values were extracted from the noneroded masks obtained from automated segmentation of the SN, SNpc, and SNpr. This approach was chosen to ensure sufficient voxel inclusion for these small substructures, as morphological erosion would have resulted in substantial loss of voxels. Analogous to the volumetric assessment, we first evaluated whether the choice of synthetic contrast used to generate the segmentation mask significantly influenced the extracted qT1 values of the total SN. Subsequently, we investigated the effects of age and sex in the total SN before investigating subregional differences.

### 2.3 Statistical Analysis

Computations were performed using JASP (version 0.19.3). Interrater reliability for manual segmentations was assessed using intraclass correlation coefficients (ICC) based on a two‐way random effects model (absolute agreement; ICC2,1) [30] and Bland–Altman analysis. To justify the use of averaged Likert scores for the statistical comparison of contrasts, an ICC for average measures (ICC2,k) indicating at least moderate agreement (≥ 0.50) was required. ICC interpretation followed the guidelines by Koo and Li [31]. Correlation analyses (Pearson's *r*) were performed to evaluate associations between variables. Two‐sided paired *t*‐tests were used to compare quantitative metrics, whereas repeated‐measures ANOVA (RM‐ANOVA) assessed differences across synthetic contrasts with post hoc Holm correction. Assumptions of normality and sphericity were checked; violations were addressed using the Greenhouse–Geisser correction (for RM‐ANOVA), Spearman's *rho* (for correlations), or Wilcoxon signed‐rank tests (for *t*‐tests). Cohen's *d* was calculated as a measure of effect size. Hierarchical regression modeled sex and age effects and a separate analysis with linear and quadratic age effects. Statistical significance was set at *p* < 0.05.

## 3 Results

### 3.1 Evaluation of Different Synthetic MRI Contrasts

#### 3.1.1 Image Quality

IR_WM yielded the highest OC, ci_T1 the highest CNR, and PSD the highest SNR, each significantly higher compared with all other synthetic contrasts (all *p* < 0.001) (Tables S2, S3, S4, Data S2), as expected from the underlying signal equations and nonlinear noise propagation. Likert scores showed moderate agreement for subjective anatomical delineation (ICC = 0.59; 95% CI [0.33, 0.74]) and overall image quality ratings (ICC = 0.64; 95% CI [0.47, 0.75]). Consequently, the averaged Likert scores served as a robust representative value per subject for all subsequent comparative analyses. Subjectively, ci_T1 was rated best for anatomical delineation relative to adjacent white matter (mean 3.75 ± 0.71; median 4, IQR [3; 4]) and overall image quality (mean 4.08 ± 0.69; median 4, IQR [4; 5]) (Tables S5 and S6, Data S2). However, visual inspection revealed that the strong contrast amplification of the ci_T1 filter led to an artificially inflated appearance of the overall SN. Furthermore, this aggressive scaling washed out subtle internal boundaries, reducing intrastructural detail between the SNpc and SNpr and occasionally obscuring the anterior border of the SN potentially due to local T1‐shortening effects in the iron‐rich SNpr. In these instances, reduced anterior SNpr boundaries were also observed in IR_WM, whereas they were correctly preserved in si^4^_PSD (Figure 2). Overall, si^4^_PSD offered a favorable trade‐off regarding objective quality metrics with sufficient subjective delineation (mean 3.19 ± 0.77; median 3, IQR [2; 4]) and perceived image quality (mean 3.55 ± 0.68; median 3, IQR [3; 4]). Results are summarized in Figure 3.

**FIGURE 2:**
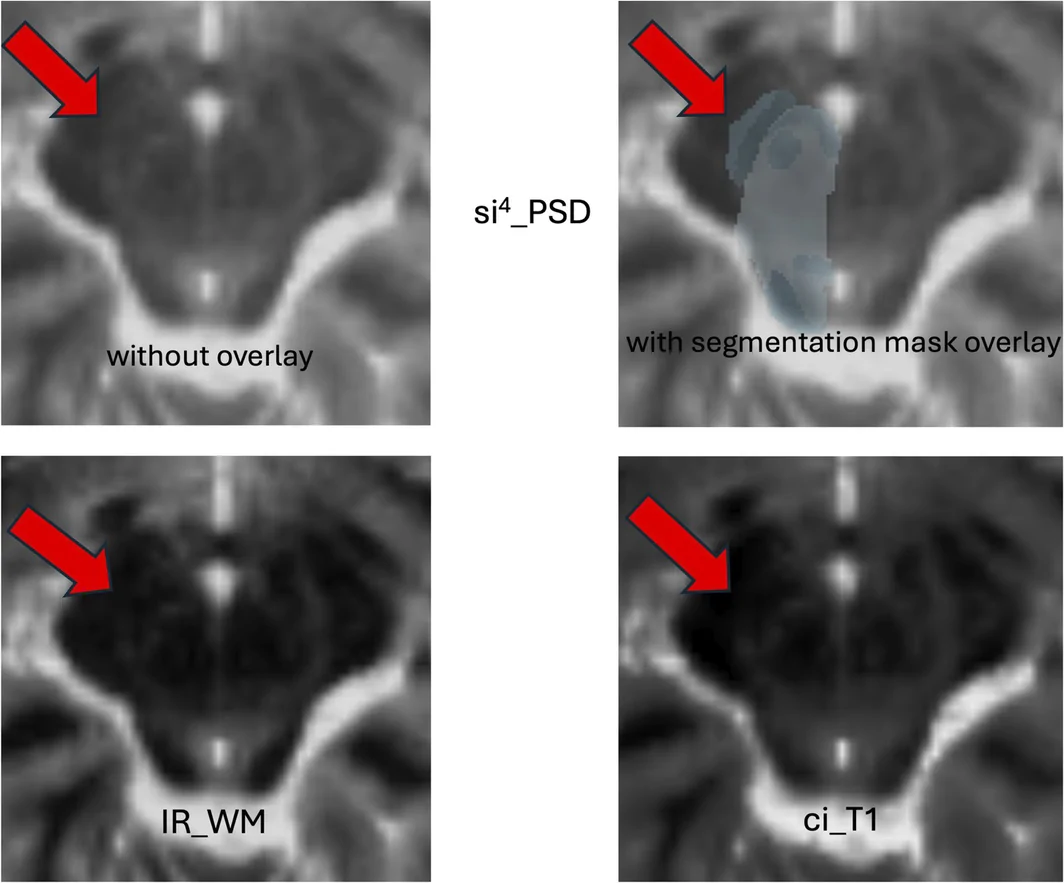
Exemplary comparison of substantia nigra (SN) delineation across different contrasts. In the axial si^4^_PSD image (top left), the anterior border of the SN is clearly visible (red arrow). The corresponding automated segmentation (top right) indicates that this border corresponds to the substantia nigra pars reticulata (SNpr). In the inversion‐recovery white matter contrast (IR_WM, bottom left), the same border is only weakly visible (red arrow), whereas in the contrast‐increased T1 (ci_T1) synthetic image (bottom right), the anterior SNpr border is no longer distinguishable (red arrow). Images are displayed without windowing. During manual volumetry, optimized windowing (white matter dark, SN bright) was applied to achieve maximal and comparable contrast across all modalities. The reduced visibility of the SNpr border in IR_WM and ci_T1 persists independently of windowing adjustments. Please note that all images were acquired and processed at their native 1‐mm^3^ isotropic resolution; the smooth appearance in this figure is merely a visual display‐level interpolation automatically applied by the viewing software (MRIcron).

**FIGURE 3:**
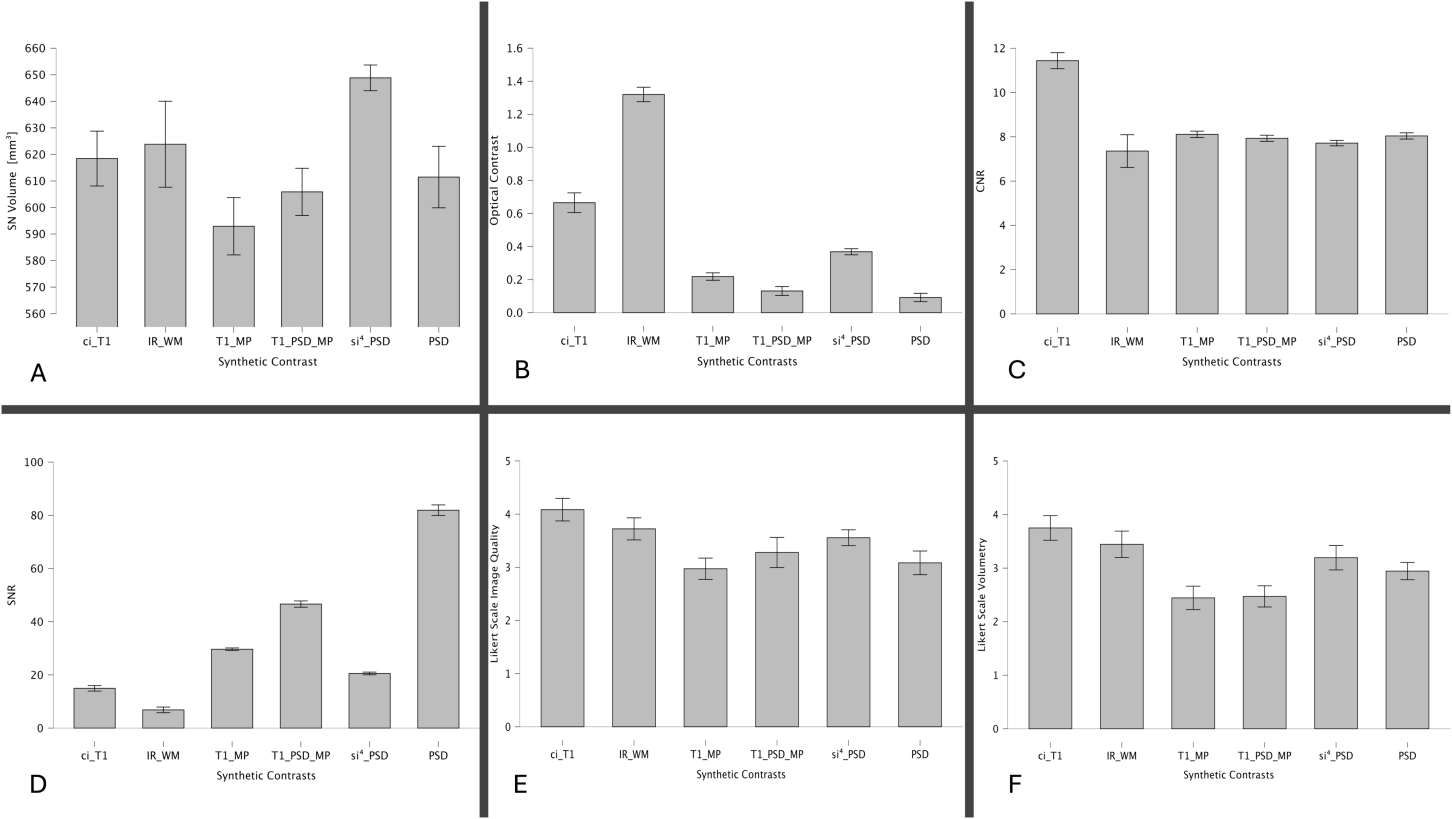
Overview of automated segmentation results and image quality metrics across synthetic contrasts. (A) Mean segmented SN volumes [mm^3^]. (B) Optical contrast. (C) Contrast‐to‐noise ratio (CNR). (D) Signal‐to‐noise ratio (SNR). (E) Subjective image quality rating (Likert scale, scores 1–5 averaged for both raters). (F) Subjective SN delineation rating (Likert scale, scores 1–5 averaged for both raters). Error bars represent 95% confidence intervals. ci = contrast‐increased; IR_WM = inversion‐recovery–based white matter (nulling); MP = MP‐RAGE‐like; MP‐RAGE = magnetization‐prepared rapid gradient echo; PSD = proton spin density; qT1 = quantitative T1; si^4^ = signal‐increased, values raised to the fourth power.

Notably, subjective ratings showed a significant negative correlation with subjects' age for all contrasts except PSD (perceived image quality: [rho ≤ −0.47, *p* < 0.05]; SN delineation: [rho ≤ −0.46, *p* < 0.05]). This indicates that the ratings were influenced by the subjects' age (Tables S7 and S8, Data S2), rather than solely reflecting the technical performance of the generated contrast. Monte Carlo‐based noise estimation compared to analytical derivation showed high consistency for synthetic PSD‐weighted contrasts in SNR and CNR calculations (*r* ≥ 0.78, *p* ≤ 0.001) (Figures S1 and S2, Data S3). However, the lower consistency observed for IR_WM (*r* ≥ 0.45, *p* ≤ 0.059), where Rician noise distribution prevails due to low SNR (6.83 ± 1.12), justified the methodological preference for Monte Carlo‐based results.

#### 3.1.2 Manual Segmentation

Poor agreement (ICC < 0.5) was observed for manual segmentation of single axial slices of the SN across all synthetic contrasts. Although overall agreement was poor, ci_T1 (ICC = 0.34; 95% CI [−0.08, 0.68]) and si^4^_PSD (ICC = 0.31; 95% CI [−0.09, 0.70]) showed the highest agreement among the evaluated contrasts. Bland–Altman analysis further indicated that manual single‐slice area measurements in si^4^_PSD yielded the narrowest 95% limits of agreement (95% LoA: 0.67 to 58.67 mm^2^; bias: 29.67 mm^2^) compared to ci_T1 (95% LoA: −35.96 to 87.74 mm^2^; bias: 25.89 mm^2^). As si^4^_PSD provided the most consistent results in blinded and randomized single‐slice manual segmentation, intrarater reliability was assessed with repeated area measurements by one of the original raters after 3 months, demonstrating good agreement (ICC = 0.75; 95% CI [0.29, 0.91]) with a slight but constant trend to smaller segmentations in Bland–Altman analysis (95% LoA: −12.34 to 28.29 mm^2^; bias: 7.94 mm^2^). For manual segmentation of the total SN, low‐to‐moderate interrater reliability was observed (ICC = 0.50; 95% CI [0.07, 0.78]), and the Bland–Altman plot revealed a volume‐dependent bias (Table S9, Data S2).

#### 3.1.3 Automated Segmentation

Automated segmentation provided more consistent results. An initial RM‐ANOVA indicated no overall statistically significant main effect of the chosen contrast type on total SN volumes (*p* = 0.254). However, an exploratory analysis specifically targeting si^4^_PSD, due to its superior agreement on manual delineation, revealed that it yielded significantly larger volumes (mean 649 ± 93 mm^3^) than all other contrasts (*p* < 0.001) and the narrowest 95% confidence interval of segmented SN sizes (Figure 3 and Table S10, Data S2). Furthermore, si^4^_PSD demonstrated the highest correlation with subject‐wise averaged volumes (*r* = 0.995, *p* < 0.001) and high correlations with the segmented volumes of all individual contrasts (*r* ≥ 0.95, *p* < 0.001) (Table S11, Data S2). In contrast to manual volumetry, repeated automated segmentation of si^4^_PSD yielded identical results across runs, indicating perfect computational reproducibility.

### 3.2 Volume Analysis

Initial analysis of manual segmentations from both raters suggested a significant linear decline in SN volume with age (rater 1: *slope* = −4.43 ± 1.53 mm^3^/year; *p* = 0.011; rater 2: *slope* = −10.57 ± 2.34 mm^3^/year; *p* < 0.001) and no significant sex differences (*p* > 0.05). However, this apparent age‐related atrophy was interpreted as a methodological artifact probably driven by a physiological effect on perceived image quality. Specifically, a significant inverse correlation between OC and the subjects' age was observed (*r* = −0.714, *p* < 0.001) (Figure S3, Data S3). This age‐dependent contrast degradation is an interesting biological finding that likely reflects progressive intrastructural changes such as iron and neuromelanin accumulation over the lifespan but critically confounded the manual anatomical delineation. Given the combination of low reliability (poor to moderate ICC) and strong susceptibility to contrast‐induced confounding, manually segmented data were considered unsuitable for further analyses.

Consequently, demographic analyses were based on automated segmentation. As RM‐ANOVA showed no significant effect of contrast type, SN volumes derived from automated segmentation (of all six contrast types) were averaged subject‐wise for further analysis (mean 617 ± 92 mm^3^). In contrast to the manual findings, age was not significantly associated with automated SN volume in this cohort (*p* = 0.442) (Figure 4). Instead, significant sex‐related differences were observed (Figure 5). Men had significantly larger SN volumes than women (mean 681 ± 72 mm^3^ vs. 552 ± 59 mm^3^; *d* = 1.85, *p* < 0.001), a difference that remained significant even after correcting for intracranial volume (ICV) (*d* = 1.06, *p* = 0.039) (Figure 5), with a median ICV of 1580 cm^3^ for men and 1430 cm^3^ for women. Lateral asymmetry was also significant, with the left SN (mean 333 ± 58 mm^3^) being larger than the right (mean 283 ± 40 mm^3^) (*d* = 0.91, *p* < 0.001) (Figure 4).

**FIGURE 4:**
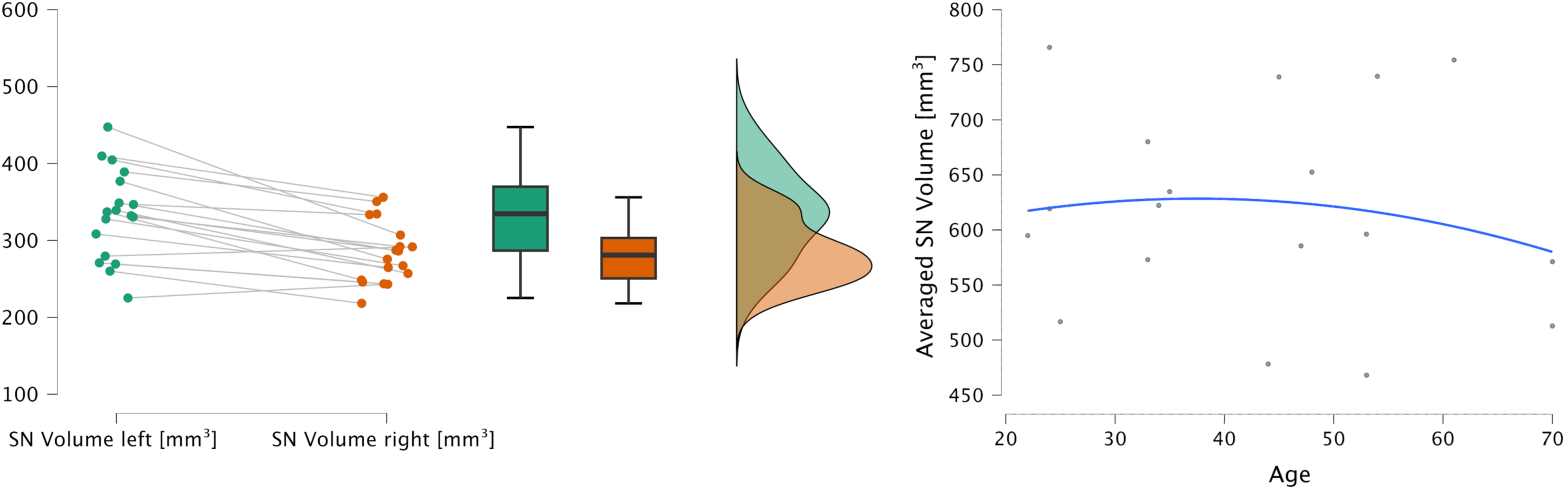
Hemispheric asymmetry and age effects on atlas‐based automated volume estimates of the substantia nigra. Left: significant mean differences in SN volumes between the right and left hemisphere (*d* = 0.91; *p* < 0.001). Individual SN volumes are shown for the left (green) and right (orange) hemispheres, with paired measurements connected by lines to illustrate intraindividual asymmetry. Boxplots represent the median and interquartile range, with whiskers indicating data dispersion. Adjacent violin plots depict the distribution density of volumes for each hemisphere, highlighting a systematic shift toward higher values in the left SN. Right: Scatterplot of segmented SN volume across age, showing no significant age‐related decline, with a fitted quadratic regression curve.

**FIGURE 5:**
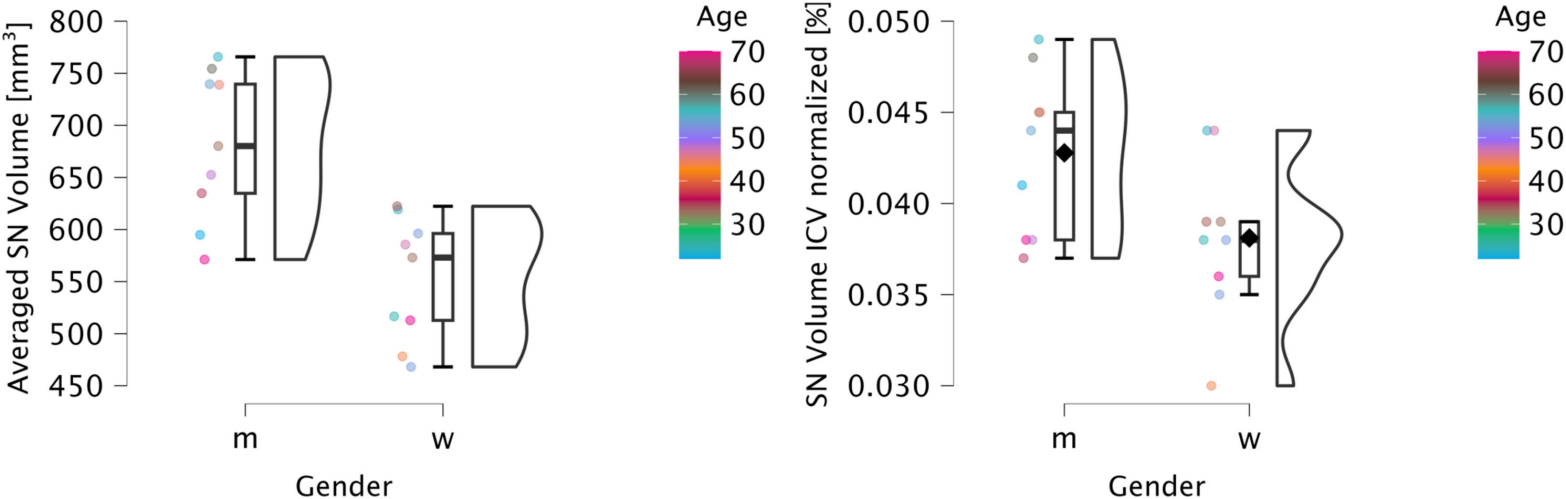
Sex‐dependent differences in atlas‐based automated volume estimates of the substantia nigra. Individual data points are shown, color‐coded by age, alongside boxplots (median and interquartile range), and violin plots illustrating distribution density. Left: Comparison of absolute SN volumes between men and women (*d* = 1.85, *p* < 0.001). Right: Comparison of SN volumes corrected for intracranial volume (ICV) between men and women (*d* = 1.06, *p* = 0.039). m = men; w = women.

Subregional analysis of subject‐wise averaged SNpc and SNpr volumes revealed demographic and asymmetric patterns identical to the total SN: neither subregion showed a significant age effect (*p* > 0.05), but both exhibited significant sex differences (*p* < 0.01) and left‐dominant asymmetry (*p* < 0.001). Finally, volumes did not differ significantly (*p* = 0.729) between the SNpc (mean 308 ± 45 mm^3^) and SNpr (mean 309 ± 49 mm^3^) subregions (Figure S4, Data S3).

### 3.3 Intrastructural qT1 Analysis

RM‐ANOVA revealed no significant main effect of the underlying synthetic contrast on the extracted mean qT1 values of the total SN (*p* > 0.05). Specifically, while the automated segmentation of different synthetic contrasts yielded minor and overall statistically nonsignificant variations in mean mask volumes, applying these slightly varying masks to the qT1 map resulted in highly consistent mean qT1 values. Given this consistent intercontrast stability, the extracted qT1 values were averaged subject‐wise for further analyses. The mean qT1 value of the total SN was 1131 ± 47 ms. Within eroded SN masks used for image quality assessment, mean qT1 values were 1157 ± 44 ms, consistent with the findings (mean 1155 ± 60 ms to 1161 ± 62 ms) reported by Baudrexel et al. [5] in healthy controls acquired using the same field strength and a comparable VFA method.

In contrast to automated segmentation‐derived volumes, sex had no significant effect on the qT1 values (*p* = 0.95). No significant differences were found between qT1 values of the left and right SN (*p* = 0.14), and no significant correlation between qT1 values and automatically segmented SN volumes (*r* = −0.30, *p* = 0.22). There was a significant subregional difference: The SNpc exhibited higher qT1 values (mean 1144 ± 55 ms) than the SNpr (mean 1117 ± 43 ms) (*d* ≥ 0.42, *p* ≤ 0.017), with the greatest differentiation achieved using the segmentation from si^4^_PSD (SNpc: mean 1144 ± 55 ms vs. SNpr: mean 1103 ± 48 ms; *d* = 0.78, *p* < 0.001) (Table S12, Data S2 and Figure S5, Data S3).

For the total SN, hierarchical regression analysis revealed that a model including both a linear (*slope* = −9.474 ± 3.751 ms/year, *p* = 0.023) and a quadratic age term (*slope* = 0.088 ± 0.041 ms/year, *p* = 0.048) significantly improved model fit over a linear‐only model (Δ*R* ^*2*^ = 14.9%, overall *p* = 0.016), suggesting a nonlinear, U‐shaped aging trend with an initial decline followed by an increase in later life. Critically, the aging patterns differed between subregions (Figure 6) and were asymmetric:

- SNpc (overall): demonstrated a significant linear decrease in qT1 with age (*slope* = −1.771 ± 0.778 ms/year, *p* = 0.037). Inclusion of a quadratic term did not improve model fit.
- SNpr (overall): Demonstrated a significant nonlinear, U‐shaped relationship with age. A model including both a linear (*slope* = −8.921 ± 3.552 ms/year, *p* = 0.024) and a quadratic age term (*slope* = 0.085 ± 0.039 ms/year, *p* = 0.046) significantly improved model fit over a linear‐only model (Δ*R* ^*2*^ = 18.9%, *p* = 0.046).
- Asymmetry: In hemispheric subanalysis, the significant *linear* decline in the SNpc was only confirmed in the *right* hemisphere (*slope* = −1.983 ± 0.845 ms/year, *p* = 0.032). Conversely, the significant *U‐shaped* pattern in the SNpr was only confirmed in the *left* hemisphere (Δ*R* ^*2*^ = 22.8%, *p* = 0.043).
- Intrastructural heterogeneity: The standard deviation of qT1 values within the SNpr increased linearly with age (*slope* = 1.056 ± 0.180 ms/year, *p* = 0.002) (Figure S6, Data S3). This metric was inversely correlated with the estimated noise level derived from error propagation of the raw VFA‐GRE signals (Data S1) calculated for SNpr (*rho* = −0.56, *p* = 0.016), suggesting it reflects true intrastructural heterogeneity rather than noise. For the SNpc, this age‐related effect on heterogeneity was not significant (*p* = 0.060).

All key results are summarized in Table 2.

**FIGURE 6:**
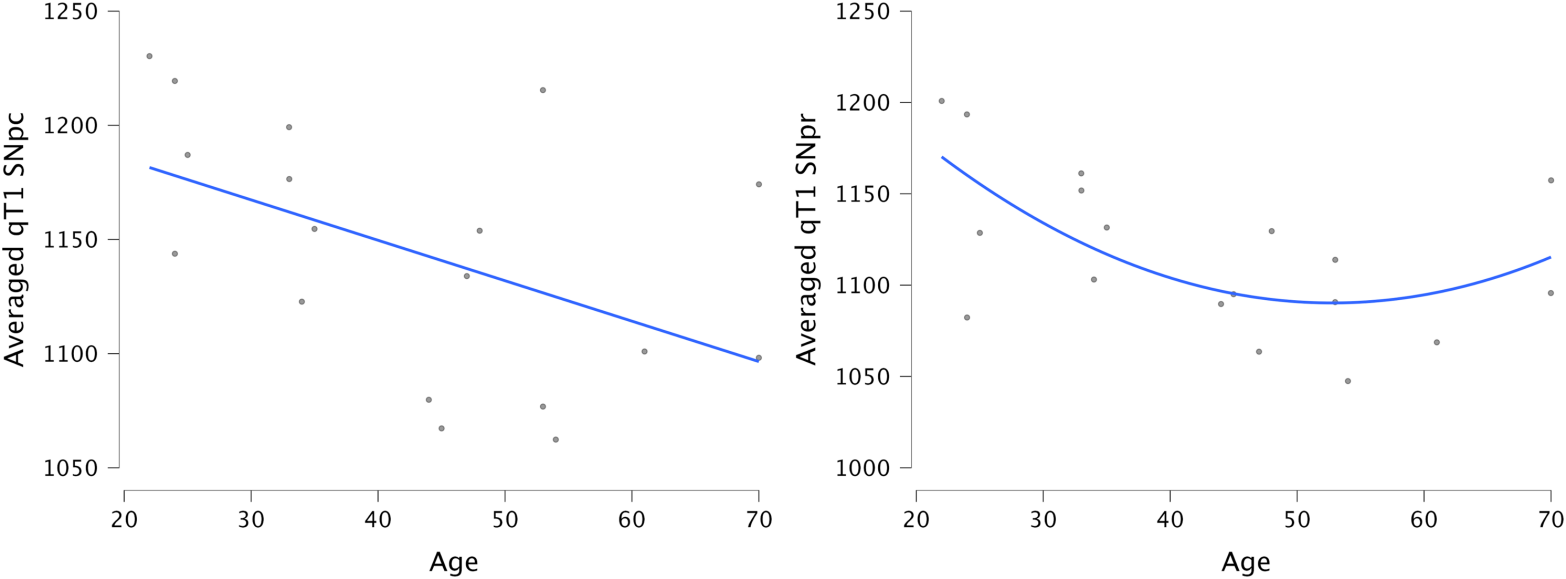
Age‐dependent trends of quantitative T1 (qT1) values in substantia nigra (SN) subregions. Scatterplots with fitted regression lines illustrating distinct aging patterns within SN subregions. Each dot represents the values of an individual subject. The SNpc (left panel) exhibits a significant linear decline in qT1 values with increasing age (*F*(1, 16) = 5.183, *p* = 0.037), with age serving as a negative predictor (*slope* = −1.771 ± 0.778 ms/year, *p* = 0.037). In contrast, the SNpr (right panel) follows a nonlinear, U‐shaped trend. Hierarchical regression revealed that the addition of a quadratic age term significantly improved the linear model fit (Δ*R* ^*2*^ = 0.189, *F*‐change (1, 15) = 4.721, *p* = 0.046). In the final quadratic model, both the linear (*slope* = −8.921 ± 3.552 ms/year, *p* = 0.024) and quadratic (*slope* = 0.085 ± 0.039 ms/year, *p* = 0.046) age terms were significant.

**TABLE 2:** Summary of key study results.

Parameter of interest	Comparison/group	Result (mean ± SD or value)	Statistic/effect size	*p*
Objective image quality				
Optical contrast	Best: IR_WM	1.32 ± 0.14	vs. others	< 0.001
Contrast‐to‐noise	Best: ci_T1	11.43 ± 2.14	vs. others	< 0.001
Signal‐to‐noise	Best: PSD	81.91 ± 5.98	vs. others	< 0.001
Subjective image quality				
Image quality rating	Best: ci_T1	4.08 ± 0.69	vs. others	< 0.01
	si^4^_PSD performance	3.55 ± 0.68	vs. T1_MP (2.97)	< 0.01
SN delineation	Best: ci_T1	3.75 ± 0.71	vs. others	< 0.01
	si^4^_PSD performance	3.19 ± 0.77	vs. T1_MP/PSD	< 0.01
Volumetry: methodology				
Manual reliability	Total SN: Interrater (si^4^_PSD)	Low‐to‐moderate agreement	ICC = 0.501	—
	Single slice: Interrater	Poor agreement (all contrasts)	ICC < 0.5	—
	Single slice: Intrarater (si^4^_PSD)	Good agreement	ICC = 0.751	
Manual bias (age)	Rater 2 volume vs. Age	*slope* = −10.57 ± 2.34 mm^3^/year (linear regression)	*R* ^*2*^ = 0.56	< 0.001
	Rater 1 volume vs. age	*slope* = −4.43 ± 1.53 mm^3^/year (linear regression)	*R* ^*2*^ = 0.287	0.011
Automated volumetry	Contrast Effect	RM‐ANOVA (main effect)	*F* = 1.41	0.254
	Exploratory post hoc	si^4^_PSD > others	*d* > 0.35	< 0.001
	Reproducibility	si^4^_PSD repeated	Identical Results	—
Volumetry: demographics				
Sex difference	Men vs. women	681 ± 72 vs. 552 ± 59 mm^3^	*d* = 1.06 (ICV‐corrected)	0.039
Hemispheric asymmetry	Left vs. right SN	333 ± 58 vs. 283 ± 40 mm^3^	*d* = 0.91	< 0.001
qT1 relaxometry				
Substructure values	SNpc vs. SNpr	1144 ± 55 vs. 1103 ± 48 ms	*d* = 0.78	< 0.001
Age trends	Total SN	U‐shaped (quadratic)	Δ*R* ^*2*^ = 14.9%	0.048
	SNpc (right)	Linear decline	*slope* = −1.98 ± 0.85 ms/year	0.032
	SNpr (left)	U‐shaped (quadratic)	Δ*R* ^*2*^ = 22.8%	0.043
Signal variability	SNpr StdDev vs. age	Linear increase	*slope* = 1.06 ± 0.18 ms/year	0.002

Abbreviations: ICC = intraclass correlation coefficients; ICV = intracranial volume; RM‐ANOVA = repeated‐measures analysis of variance, SN = Substantia nigra; SNpc = substantia nigra pars compacta; SNpr = substantia nigra pars reticulata.

To investigate whether we can visualize the observed qT1 differences of the SNpr and SNpc, we performed an exploratory analysis with heatmaps of normalized qT1 maps of the SN. The inter‐individual and group‐level analysis revealed clustered hyperintensities in the caudal SN, consistent with the anatomical location of Nigrosome 1, the region with the highest density of dopaminergic neurons [1] (Figure 7).

**FIGURE 7:**
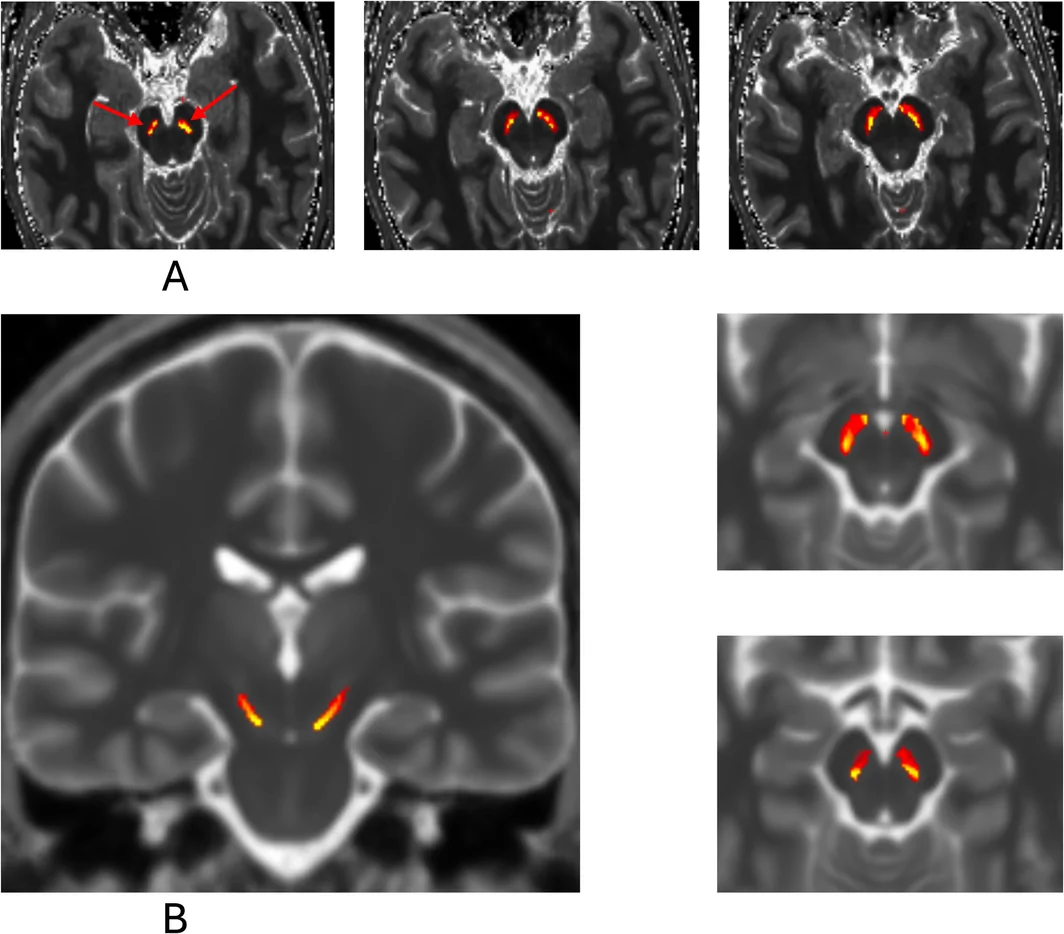
Visualization of nigrosomal substructures. (A) qT1 map overlaid with normalized qT1 heatmaps of the caudal, medial and cranial SN, demonstrating clustered hyperintensities in a healthy volunteer. The left image shows the caudal region, with clusters of relatively high qT1, possibly corresponding to Nigrosome 1 (red arrow), which matches the presentation of the Nigrosome 1 sign reported by Cheng et al. [32] in SWI. (B) Group‐level trend analysis confirming the consistency of this “qT1‐hotspot” across the cohort, likely reflecting high neuronal density. Right axial images display group‐level hotspots of relatively high qT1 values aligning with MTC clusters as described by Langley et al. [33] in the caudal and medial SN.

## 4 Discussion

This study demonstrates that a qMRI‐based approach, combining qT1 mapping with pPSD‐derived, nonlinearly enhanced synthetic contrast si^4^_PSD, improves the visual delineation and consistency of quantitative assessment of SN when paired with automated segmentation. Importantly, this framework enables simultaneous assessment of intrastructural properties via qT1, and volumetric analysis based on qT1‐derived synthetic contrasts via pPSD. Conversely, a central finding is the limitation of manual segmentation, where age‐dependent contrast reduction is misinterpreted as atrophy—a so‐called circularity of error, as previously described [26]. Automated, atlas‐based segmentation overcame this, providing SN volume estimates that were not significantly associated with age in this cohort, supporting their suitability for reliable quantification. Prior to NextBrain, automated SN segmentation was not feasible in standard segmentation pipelines mainly using conventional MP‐RAGE imaging [22]. Because the SN exhibits only minimal contrast on standard T1‐weighted MRI, it was historically excluded from atlases like FreeSurfer or FSL‐FIRST; instead existing methods relied on susceptibility contrasts or diffusion tensor imaging [22, 34].

Notably, the chosen VFA protocol provides optimal noise performance near the local Ernst angle of approximately 9.8° (corresponding to a T1 of ~890 ms). This ensures that error progression remains minimal in the T1 range of WM in the midbrain, making this specific acquisition highly suitable for the purpose of this study. For a detailed mathematical description of the noise progression from the raw GRE images to the qT1 maps and the derived synthetic contrasts, we refer to Data S1.

Paradoxically, the ci_T1 contrast, which achieved the highest CNR and subjective quality ratings, led to inaccuracies in the delineation of SN boundaries. The selective amplification approach, based on the T1‐dependent Fermi filter as detailed in Section 2, homogenized signal within the presumed SN region, obscuring natural transition zones and leading to a loss of the anterior SNpr boundary in some cases (Figure 2).

These findings emphasize that the optimization of tissue contrast is inherently dependent on the target structure. High values in individual image quality metrics, such as CNR or OC, do not necessarily translate into more accurate volume quantification. In contrast, for the extraction of quantitative tissue parameters such as qT1, higher SNR and CNR remain beneficial for improving measurement precision [24]. Optimization of a single metric may be detrimental when it alters intrinsic signal heterogeneity or boundary definition or is accompanied by massive reductions in SNR, thereby compromising the reliability of downstream analyses. Similar trade‐offs between CNR, SNR, and segmentation performance have been reported by Büeler et al. [35] in the context of spinal cord segmentation.

The si^4^_PSD contrast emerged as a highly consistent basis for automated segmentation, yielding the most stable volumetric estimates. Notably, it also resulted in the largest SN volumes. Although larger volumes do not inherently guarantee higher accuracy, improved structural definition is further corroborated by the quantitative analysis: Although all contrasts allowed for a significant differentiation between SNpc and SNpr, segmentation based on si^4^_PSD yielded the most pronounced separation. This matches recent findings using the PENCIL technique, where PSD‐enhanced contrasts also yielded larger SN volumes in healthy controls and were suggested to provide improved specificity for monitoring disease progression of PD compared with conventional NM‐MRI [14].

Although multiparametric approaches combining MT saturated and PSD signals have proven valuable for segmenting broad monoaminergic brain stem clusters, isolating the SN with its specific subregional histological architecture including SNpc and SNpr requires a highly targeted approach [36]. Our findings support the notion that PSD contributes substantially to the intrinsic contrast of neuromelanin‐containing structures [7, 10] and extend this concept using a synthetic contrast derived from a qT1‐map via pPSD. Furthermore, the potential influence of T2* on pPSD estimation must be considered. Because our calculation relies on an empirical inverse relationship without explicit T2* correction, local T2* shortening effects (e.g., physiological iron accumulation) decrease effective qT1, artificially lowering the calculated pPSD value. Although providing useful contrast in healthy controls, pathological iron accumulation in PD could further decrease apparent pPSD, potentially degrading contrast and underestimating segmented volumes. The nonlinear signal increase (raising pPSD values to the fourth power) enhanced the hyperintense SN signal relative to the adjacent WM while preserving the natural signal hierarchy and subtle texture information required for atlas‐based segmentation. Although this operation reduces the nominal SNR by a factor of 4, the initially high SNR of the pPSD map ensures that it remains sufficient for reproducible segmentation.

The observed qT1 findings indicate differential aging patterns within SN subregions in vivo. The significantly higher qT1 in the SNpc compared with the SNpr may reflect underlying histological differences, including a higher density of large dopaminergic, neuromelanin‐containing neurons in the SNpc, versus a higher iron content and different cellular organization in the GABAergic SNpr [37]. The distinct aging patterns are noteworthy. The linear qT1 decline within the SNpc, significant in the right hemisphere, is consistent with progressive physiological accumulation of qT1‐shortening substances, potentially neuromelanin [38], which is formed as a by‐product of dopamine metabolism. The U‐shaped trend in the SNpr, significant in the left hemisphere, suggests a more complex model: an initial phase of iron accumulation (decreasing qT1) until midlife [38], followed by age‐related intrastructural changes (e.g., demyelination, altered water content) that counteract and eventually outweigh this effect, leading to an increase in qT1 in later life [39]. The concomitant age‐related increase in qT1 heterogeneity within the SNpr, independent of noise, further supports a progressive loss of intrastructural homogeneity. Notably, qT1 values are sensitive to tissue intrastructural changes; however, these alterations are not histopathologically specific. Consequently, effective qT1 variations can indicate underlying processes (aggregating several effects including iron accumulation and MT effects), but they do not allow definitive differentiation without corroborating histological or multimodal evidence. This asymmetry, preferential linear aging in the right SNpc versus nonlinear aging in the left SNpr, represents, to our knowledge, the first in vivo evidence of lateralized intrastructural time course of aging within the healthy SN. This inherent biological heterogeneity may provide a physiological basis for lateralization in PD [5, 40, 41].

Manual, intensity‐based volumetry can be inherently confounded by physiological aging processes (e.g., iron/neuromelanin accumulation affecting contrast), potentially leading to the misinterpretation of signal loss as atrophy [34]. In contrast, our results show that when volume is assessed independently via a probabilistic atlas, it remains stable, whereas qT1 values reveal distinct intrastructural patterns. Thus, macrostructural volumetric measures and intrastructural quantitative metrics should be considered as separate, complementary biomarkers rather than interdependent variables. The reliability of segmentation using the probabilistic NextBrain atlas and subsequent qT1 analysis is further corroborated by our results, showing that qT1 values extracted from masks generated across all six different synthetic contrast weightings remained highly consistent [22].

We identified a significant lateral asymmetry (left > right), aligning with results from large‐scale diffusion MRI studies [42] and histological findings [43]. Regarding sex differences, we observed significantly larger SN volumes in men compared to women, a difference that persisted even after correction for intracranial volume. Although the latest meta‐analysis suggests that most sex dimorphisms are eliminated by size correction [44], a previous meta‐analysis reported region‐specific sex discrepancies in subcortical gray matter volumes [45] and an automated segmentation study found gender‐specific patterns in SNpc size for healthy controls [46]. Given the known sex differences in PD prevalence, symptom presentation, and progression [47, 48, 49], further establishing these normative baseline differences in future studies is crucial for accurate disease monitoring.

An exploratory finding was that intraindividual scaling of qT1 values to the qT1 range of the segmented SN enabled visualization of nigrosomal substructures. This relative contrast visualization consistently revealed clusters with elevated qT1 values in the caudal SN, likely corresponding to Nigrosome 1. The group‐level localization aligns with magnetization transfer contrast (MTC) clusters described by Langley et al. [33], whereas the observed inter‐individual variability matches findings by Cheng et al. [32]. These “T1‐hotspots” likely reflect a high density of dopaminergic neurons with lower iron content compared to the surrounding SN [37]. The loss of this hotspot could potentially serve as an earlier biomarker than global volume reduction [1], consistent with qT1 reductions observed in PD by Baudrexel et al. [5].

Finally, it should be noted that all synthetic contrasts compared in this study were derived solely from qT1 maps, as the goal was to present a method that is broadly applicable even if the quantitative MRI protocol only comprises T1 mapping. However, if a dedicated multiparametric protocol is used that yields actual PSD maps in addition, the direct construction of synthetic data should yield similar or further improved visual results and holds potential for future studies. Still, great care must be taken that these maps are completely corrected for any RF coil bias, as residual nonuniformities may be strongly enhanced when calculating the fourth power of PSD, making the generated synthetic contrasts highly susceptible to artifacts [50].

### 4.1 Limitations

One of the primary limitations of this study is the lack of a subject‐specific histological ground truth and a test–retest reliability assessment for qT1 and volumetric measurements on the same volunteers due to the retrospective analysis. We cannot definitively verify that the segmented volumes in our cohort correspond exactly to the anatomical distribution in vivo. However, a key methodological advantage of this study is the use of the NextBrain atlas, which incorporates cytoarchitectonic data derived from postmortem histology, thereby mitigating the lack of individual histological validation [18]. Second, the study is limited by its small, cross‐sectional cohort (*n* = 18), which precludes longitudinal assessments and robust analyses of age‐by‐sex interactions. Third, manual segmentation is inherently dependent on rater expertise; although raters were trained and familiar with neuroradiological anatomy, they were not expert readers, which may have contributed to the observed low interrater reliability and underscores the need for user‐independent automated approaches. Fourth, methodologically, our approach relies on a derivation chain (acquisition: VFA‐GRE; postprocessing: qT1 maps and pPSD maps → synthetic images) susceptible to error propagation. Fifth, it should be noted that the qT1‐derived pPSD maps were created solely for the purpose of constructing synthetic image weightings with an improved contrast for SN segmentation. However, they should not be used for other purposes (such as the comparison of quantitative PSD values between healthy controls and individuals with PD) as they do not constitute actual PSD maps: In particular, they are not corrected for T2* effects and rely on an empirical inverse relationship between T1 and PSD. Although this approximation allows for efficient receive coil profile correction [19], it implies that pathological processes affecting T1 relaxation could mistakenly alter the apparent PSD contrast [21], potentially resulting in lower segmented volumes, thereby confounding interpretation in disease states. Last, we did not directly compare our results with standard NM‐MRI, quantitative susceptibility mapping, or other vendor‐independent protocols like STAGE, PENCIL, or similar new approaches, which could provide complementary quantitative information [13, 51]. Finally, the exploratory visualization of nigrosomal clusters requires validation against established techniques such as high‐resolution 7 T SWI [52].

## 5 Conclusion

Synthetic nonlinear pPSD signal enhancement (si^4^_PSD) enables highly consistent, automated segmentation of the SN, overcoming age‐related contrast variability and rater‐dependent bias inherent to manual methods. The qT1 tissue data reveal distinct, asymmetrically evolving aging patterns across the SN subregions SNpr and SNpc. This qT1‐based mapping and imaging framework provides a promising avenue for improving the objective diagnosis and monitoring of PD as a targeted simplification of previous multimodal protocols that require separate acquisitions for volumetry, iron mapping, and NM‐sensitive MRI and are limited by prolonged acquisition times with increased susceptibility to motion artifacts (particularly in PD populations) together with intersequence coregistration challenges [53].

## Author Contributions

**L.v.E.** performed the data analysis, manual and automated segmentation, generated the exploratory normalized SN heatmaps, contributed to the noise simulation and error propagation, and drafted the manuscript. **L.K**. performed the manual segmentation and contributed to the critical revision of the manuscript. **D.C.T.** provided methodological advice on various topics and substantially contributed to the critical revision of the manuscript. **E.H.** contributed to the critical revision of the manuscript. **R.D**. generated the synthetic datasets and qT1 maps, designed the noise simulation, assisted with the error propagation, and substantially contributed to the critical revision of the manuscript. **C.T.A**. supervised the project, provided extensive guidance, and contributed to the critical revision of the manuscript.

## Funding

Open Access funding enabled and organized by Projekt DEAL. The authors declare that no funds, grants, or other support were received during the preparation of this manuscript.

## Conflicts of Interest

The authors declare no conflicts of interest.

## Supporting information


**Data S1:** Supporting information.

**Table S1:** 5‐point Likert scale criteria used for evaluating synthetic MRI datasets.
**Table S2:** ANOVA post hoc comparisons of SN optical contrast (OC).
**Table S3:** ANOVA post hoc comparisons of SN contrast‐to‐noise ratio (CNR).
**Table S4:** ANOVA post hoc comparisons of SN signal‐to‐noise ratio (SNR).
**Table S5:** ANOVA post hoc comparisons of rated image quality.
**Table S6:** ANOVA post hoc comparisons of rated SN delineation.
**Table S7:** Correlation of image quality rating and age.
**Table S8:** Correlation of SN delineation rating and age.
**Table S9:** ICC and Bland–Altman analyses.
**Table S10:** ANOVA post hoc comparisons of SN segmentation.
**Table S11:** Correlation of segmented SN volumes.
**Table S12:** qT1 differences between SNpc and SNpr.

**Figure S1:** Comparison of analytical and simulated contrast‐to‐noise (CNR) values of the midbrain across different synthetic image contrasts.
**Figure S2:** Comparison of analytical and simulated signal‐to‐noise ratio (SNR) values of the midbrain across different synthetic image contrasts.
**Figure S3:** Association of age and optical contrast (OC) in the midbrain.
**Figure S4:** Comparison of segmented volumes of the substantia nigra pars reticulata (SNpr) and substantia nigra pars compacta (SNpc).
**Figure S5:** Comparison of quantitative T1 (qT1) values between the substantia nigra pars reticulata (SNpr) and substantia nigra pars compacta (SNpc).
**Figure S6:** Age‐related changes in intrastructural heterogeneity within substantia nigra subregions.

## Data Availability

The data that support the findings of this study are available from the senior author upon reasonable request.
