# Supplementary material for: Volumetric and Intrastructural Characterization of the Substantia Nigra Using Optimized Synthetic MR Images Derived From Quantitative T1 Maps at 3 T
Source: NMR Biomed. 2026 Oct 8;39(11):e70421. doi: 10.1002/nbm.70421 (PMC13650174; doi:10.1002/nbm.70421)
Supplement: Supplementary file 1 [file NBM-39-e70421-s001.docx]

**Volumetric and Intrastructural Characterization of the Substantia Nigra using Optimized Synthetic MR Images derived from Quantitative T1 Maps at 3T**

**Supplementary S3 (Supplementary Figures)**

**Figure S1: Comparison of analytical and simulated contrast-to-noise (CNR) values of the midbrain across different synthetic image contrasts.**

The table (top right) summarizes descriptive statistics (mean, standard deviation, minimum, and maximum) of analytical (a) and simulated (s) CNR values for si^4^_PSD, PSD and IR_WM contrasts. For the other synthetic datasets analytical error propagation was considered too complex. Scatter plots show the relationship between simulated (s) and analytical (a) CNR values for the substantia nigra and adjacent white matter in the crus cerebri of the midbrain in PSD (bottom left), IR_WM (bottom right), and si^4^_PSD (top left) synthetic contrasts. Pearson correlation coefficients (*r*) and corresponding 95% confidence intervals are indicated for each contrast. A strong agreement between analytical and simulated CNR is observed for si^4^_PSD (*r* = 0.991) and PSD (*r* = 0.913), whereas IR_WM shows only low agreement (*r* = 0.453).

**Figure S2: Comparison of analytical and simulated signal-to-noise ratio (SNR) values of the midbrain across different synthetic image contrasts.**

The table (top right) summarizes descriptive statistics (mean, standard deviation, minimum, and maximum) of analytical (a) and simulated (s) SNR values for si^4^_PSD, PSD and IR_WM contrasts. For the other synthetic datasets analytical error propagation was considered too complex. Scatter plots show the relationship between simulated and analytical SNR values for the substantia nigra and adjacent white matter in the crus cerebri in PSD (bottom left), IR_WM (bottom right), and si^4^_PSD (top left) synthetic contrasts. Pearson correlation coefficients (*r*) and corresponding 95% confidence intervals are indicated for each contrast. A strong agreement between analytical and simulated SNR is observed for PSD (*r* = 0.948), whereas si^4^_PSD (*r* = 0.782) shows good and IR_WM (*r* = 0.618) moderate agreement.

**Figure S3: Association of Age and Optical Contrast (OC) in the midbrain.**

Scatter plot shows the relationship between age and OC for the si^4^_PSD contrast, including the linear regression line (*r*) with corresponding 95% confidence interval (CI). A significant negative correlation between OC and age is observed (*r* = −0.714, *p* < 0.001). Similar significant associations were observed across all other datasets.

**Figure S4: Comparison of segmented volumes of the substantia nigra pars reticulata (SNpr) and substantia nigra pars compacta (SNpc).**

Paired data points (left) show individual subject volumes for SNpr and SNpc, with connecting lines indicating within-subject comparisons. Boxplots (center) summarize the distribution of volumes (median, interquartile range, and range), while violin plots (right) illustrate the underlying distribution density for both regions. No significant difference in mean volume between SNpr and SNpc is observed.

**Figure S5: Comparison of quantitative T1 (qT1) values between the substantia nigra pars reticulata (SNpr) and substantia nigra pars compacta (SNpc).**

Paired data points (left) show intra-individual mean qT1 values for SNpr and SNpc, with connecting lines indicating subject-wise differences. Boxplots (center) summarize the distribution of qT1 values (median, interquartile range, and range), while violin plots (right) illustrate the underlying distribution density for both regions. SNpc exhibits consistently higher mean qT1 values than SNpr, with a significant intra-individual difference.

**Figure S6: Age-related changes in intrastructural heterogeneity within substantia nigra subregions.**

Scatter plots with fitted regression lines illustrate the relationship between age and the standard deviation (SD) of quantitative T1 (qT1) values for the substantia nigra pars compacta (SNpc, left) and the substantia nigra pars reticulata (SNpr, right). Each dot represents the values of an individual subject. The SNpc exhibits only a moderate trend of increasing qT1 SD with age, statistically non-significant in regression analysis (*p* = 0.060), possibly due to lifelong neuromelanin accumulation. Conversely, the SNpr demonstrates a pronounced and significant linear increase in qT1 variance (*slope* = 1.056 ± 0.180, *p* = 0.002), characterized by a visually distinct rise in SD starting around the fourth decade of life, coinciding with the plateauing of iron accumulation and the onset of normal age-related intrastructural changes. Independent of estimated noise levels, this significant trend in the SNpr indicates a progressive, age-related loss of intrastructural homogeneity specific to this subregion.
