# Supplementary material for: Volumetric and Intrastructural Characterization of the Substantia Nigra Using Optimized Synthetic MR Images Derived From Quantitative T1 Maps at 3 T
Source: NMR Biomed. 2026 Oct 8;39(11):e70421. doi: 10.1002/nbm.70421 (PMC13650174; doi:10.1002/nbm.70421)
Supplement: Supplementary file 2 [file NBM-39-e70421-s002.docx]

**Volumetric and Intrastructural Characterization of the Substantia Nigra using Optimized Synthetic MR Images derived from Quantitative T1 Maps at 3T**

**Supplementary S2 (Supplementary Tables)**

**Table S1: 5-point Likert scale criteria used for evaluating synthetic MRI datasets.**

Left: Criteria for SN delineation during manual volumetry. Right: Criteria for evaluating general image quality in the SN region.

**Table S2: ANOVA Post-Hoc Comparisons of SN Optical Contrast (OC).**

All datasets differ significantly in OC. IR WM exhibits the highest mean difference.

**Table S3: ANOVA Post-Hoc Comparisons of SN Contrast-to-Noise Ratio (CNR).**

Enhanced T1 differs significantly in CNR from other datasets, showing the highest mean difference. IR WM does not differ significantly from the remaining datasets.

**Table S4: ANOVA Post-Hoc Comparisons of SN Signal-to-Noise Ratio (SNR).**

All datasets differ significantly in SNR. PSD shows the highest mean difference compared to other synthetic image contrasts.

**Table S5: ANOVA Post-Hoc Comparisons of Rated Image Quality.**

Ci_T1 has the significantly highest mean difference in image quality ratings compared to other datasets.

**Table S6: ANOVA Post-Hoc Comparisons of Rated SN Delineation.**

Ci_T1 has the significantly highest mean difference in SN delineation ratings compared to other datasets.

**Table S7: Correlation of Image Quality Rating and Age.**

PSD shows no significant correlation between image quality rating and age. All other datasets show a significant negative association.

**Table S8: Correlation of SN Delineation Rating and Age.** PSD and ci_T1 show no significant correlation between SN delineation rating and age. However, a non-significant negative trend was observed for Enhanced T1. All other datasets show a significant negative association.

**Table S9: ICC and Bland-Altman Analyses.**

Top: ICC results for inter-rater reliability. Middle: Bland-Altman plots showing agreement between raters. Bottom: Detailed Bland-Altman tables. Left column: Whole SN volumetry. Middle column: Selected axial slice (exemplary for si^4^_PSD). Right column: Intra-rater reliability for repeated manual volumetry (si^4^_PSD).

**Table S10: ANOVA Post-Hoc Comparisons of SN Segmentation.**

Post-Hoc tests indicating that si^4^_PSD yielded significantly larger segmented SN volumes compared to other datasets (although the overall RM-ANOVA was not significant).

**Table S11: Correlation of Segmented SN Volumes.**

Correlation matrix showing significant positive associations between all segmented SN volumes. Si^4^_PSD shows high correlation with other contrasts and highest with subject-wise averaged volume.

**Table S12: qT1 Differences between SNpc and SNpr.**

QT1 values of SNpc and SNpr, averaged subject-wise across all dataset segmentations, show a significant difference. This is most pronounced in qT1 values extracted via si^4^_PSD segmentation.
