# Supplementary material for: Volumetric and Intrastructural Characterization of the Substantia Nigra Using Optimized Synthetic MR Images Derived From Quantitative T1 Maps at 3 T
Source: NMR Biomed. 2026 Oct 8;39(11):e70421. doi: 10.1002/nbm.70421 (PMC13650174; doi:10.1002/nbm.70421)
Supplement: Supplementary file 3 [file NBM-39-e70421-s003.docx]

**Volumetric and Intrastructural Characterization of the Substantia Nigra using Optimized Synthetic MR Images derived from Quantitative T1 Maps at 3T**

**Supplementary S1 (Supplementary Equations)**

**Table of Contents**

[Simulation	3](#_Toc225924572)

[Determination of Image Noise	3](#_Toc225924573)

[Noise Calculation for the qT1 Map	3](#_Toc225924574)

[Signal Equations of the Synthetic Contrasts	5](#_Toc225924575)

[Proton Spin Density (PSD)	5](#_Toc225924576)

[Potentiated Proton Spin Density (si^4^_PSD)	5](#_Toc225924577)

[Inversion-Recovery with WM Nulling (IR_WM)	6](#_Toc225924578)

[Contrast-Increased qT1 Map (ci_T1)	6](#_Toc225924579)

[MP-RAGE (T1_PSD_MP & T1_MP)	7](#_Toc225924580)

[(i) Acquisition Parameters	7](#_Toc225924581)

[(ii) Sequence and Timing Parameters	7](#_Toc225924582)

[(iii) Relaxation Parameters	8](#_Toc225924583)

[(iv) Signal Calculation	8](#_Toc225924584)

[Image Quality	9](#_Toc225924585)

[Optical Contrast (OC)	9](#_Toc225924586)

[Contrast-to-Noise Ratio (CNR)	9](#_Toc225924587)

[Signal-to-Noise Ratio (SNR)	9](#_Toc225924588)

[References	10](#_Toc225924589)

# **Simulation**

The objective evaluation of the synthetic contrasts is based on three metrics for assessing technical image quality. To calculate these, a small slice of a qT1 map^1^ is simulated for two defined regions A and B based on T1 relaxation times (qT1) measured in subjects, as well as various synthetic datasets derived from these using pseudo-Proton Spin Density (pPSD)^2^. The methodology for defining and calculating the following metrics and for determining image noise follows the principles described in Preibisch & Deichmann^3^, Baudrexel et al.^4^, and Nöth et al.^5^.

## **Determination of Image Noise**

To determine the noise of the VFA-GRE datasets ($\sigma_{S}$), an approach based on Nöth et al.^5^, was adapted for the simulation: First, the standard deviation was measured using a circular region-of-interest (ROI) in a homogeneous background area outside the head across all subjects, and the results were subsequently averaged. Second, as these data are derived from modulus images, the noise was obtained by dividing this measured background standard deviation by a Rayleigh factor of 0.701. Finally, this resulting mean noise level was assumed to be identical for both VFA-GRE datasets in subsequent calculations. To estimate the derived noise level a simulation with one slice of a qT1 map with 500x500 pixels is calculated for each of the two regions A and B to be compared. Random values from a Gaussian distribution are added to the respective mean qT1 values. The standard deviation of this normal distribution corresponds to the previously calculated noise values. All further synthetic contrasts (e.g., Inversion-Recovery) are derived from these noisy slices of the qT1 maps for regions A and B. The noise of all simulated datasets ($\sigma_{A},\sigma_{B}$) for the SNR and CNR calculation is now determined empirically and corresponds to the standard deviation within the entire simulated slice for the respective region $\sigma_{A}=\text{std}(\text{Signal values in Region A})$ and $\sigma_{B}=\text{std}(\text{Signal values in Region B})$.

## **Noise Calculation for the qT1 Map**

For the simulation, the noise in the qT1 map ($\sigma_{T_{1}}$) was calculated based on the method of Preibisch & Deichmann^3^ from the underlying acquired variable flip angle gradient echo (GRE-VFA) datasets considering error propagation:

$$\sigma_{T_{1}}=\sqrt{\left( \frac{\partial T_{1}}{\partial S_{1}} \right)^{2}\sigma_{S}^{2}+\left( \frac{\partial T_{1}}{\partial S_{2}} \right)^{2}\sigma_{S}^{2}}$$

(1)

Here, the partial derivatives of the quantitative $T_{1}$ value with respect to the input signals ($S_{1},S_{2}$) are given by:

$$\begin{aligned} \frac{\partial T_{1}}{\partial S_{1}}=\frac{T_{1}^{2}}{TR}\cdot\frac{S_{2}\cdot\sin\left( \alpha_{1}^{'} \right)\sin\left( \alpha_{2}^{'} \right)\left( \cos\left( \alpha_{1}^{'} \right)-\cos\left( \alpha_{2}^{'} \right) \right)}{A_{1}\cdot A_{2}} \\ \frac{\partial T_{1}}{\partial S_{2}}&=\frac{T_{1}^{2}}{TR}\cdot\frac{-S_{1}\cdot sin(\alpha_{1}^{'})sin(\alpha_{2}^{'})(cos(\alpha_{1}^{'})-cos(\alpha_{2}^{'}))}{A_{1}\cdot A_{2}} \end{aligned}$$

(2)

The terms $A_{1}$ and $A_{2}$ used in the denominators are defined as:

$$\begin{aligned} A_{1}&=S_{2}sin(\alpha_{1}^{'})cos(\alpha_{2}^{'})-S_{1}sin(\alpha_{2}^{'})cos(\alpha_{1}^{'}) \\ A_{2}&=S_{2}sin(\alpha_{1}^{'})-S_{1}sin(\alpha_{2}^{'}) \end{aligned}$$

(3)

The corrected flip angles ($\alpha^{'}$) used in the derivatives are based on several correction steps. The actual flip angle $\alpha_{\text{actual}}$ is first corrected for $B_{1}$ inhomogeneities:

$$\alpha_{\text{actual}}=C_{RF}\cdot\alpha_{\text{nominal}}$$

(4)

Here, *C_RF_* represents the ratio of the actual to the nominal B_1_ amplitude. Subsequently, according to Baudrexel et al.^4^, a correction factor $C$ for insufficient radiofrequency (RF) spoiling of residual transverse magnetization is applied, which is calculated via a 5th order 2D polynomial:

$$C=\sum_{k,l=0}^{k+l\leq5} P_{k,l}\cdot\alpha_{\text{actual}}^{k}\cdot TR^{l}$$

(5)

This results in the effective "apparent" flip angles $\alpha_{1,2}^{'}$:

$$\alpha^{'}=C\cdot\alpha_{\text{actual}}$$

(6)

The parameters used in the equations are defined as follows:

- $\sigma_{T_{1}}$: The calculated noise in the qT1 map.
- $T_{1}$: The mean qT1 value in the corresponding region.
- $S_{1,2}$: The signal intensities in the two acquired GRE-VFA datasets.
- $\sigma_{S}$: The noise in the input datasets (assumed to be identical for both as they were acquired with the same FoV, matrix size and receiver bandwidth).
- $TR$: The repetition time used (16.4 ms).
- $\alpha_{\text{nominal}}$: The flip angles used in the VFA-GRE datasets ($\alpha_{1}=4^{\circ},\alpha_{2}={24}^{\circ}$).
- $C_{RF}$: The correction factor from the $B_{1}$ map. For the simulation, the mean factors for gray ($C_{RF}=1.09$) and white matter ($C_{RF}=1.00$) were used^3^.
- $P_{k,l}$: The polynomial coefficients from Baudrexel et al.^4^. These coefficients are valid for a radiofrequency spoiling phase increment of $\Delta\phi={50}^{\circ}$.

**Signal Equations of the Synthetic Contrasts**

As a supplement to the methodology, the mathematical foundations of the generated synthetic datasets are explained in this section. For the contrasts Proton Spin Density (PSD), potentiated PSD (si^4^_PSD), and Inversion-Recovery with White Matter signal nulling (IR_WM), the analytical calculation of noise from the qT1 map is additionally presented. For the synthetic Magnetization Prepared Rapid Gradient Echo (MP-RAGE) as well as the qT1 map with contrast increase of the SN (ci_T1), only the signal equations are listed.

## **Proton Spin Density (PSD)**

The calculation is based on the qT1 map and is approximated as pseudo-Proton Spin Density (pPSD) using the method of Volz et al.^2^, grounded in historically established principals^6^:

$$pPSD(T_{1})=\frac{C}{A+\frac{B}{T_{1}}}$$

(7)

Where:

- $T_{1}$: T1 relaxation time in the corresponding region [ms].
- $C$ = 100 (to scale pPSD values to about 100% in CSF)
- $A$ = 0.858
- $B$ = 522

According to the Gaussian error propagation law, the noise in the pPSD map ($\sigma_{pPSD}$) can be calculated from the noise of the underlying qT1 map ($\sigma_{T_{1}}$):

$$\sigma_{pPSD}=\left| \frac{\partial pPSD(T_{1})}{\partial T_{1}} \right|\cdot\sigma_{T_{1}}$$

(8)

Derivative with respect to $T_{1}$:

$$\frac{\partial pPSD(T_{1})}{\partial T_{1}}=\frac{C\cdot B}{(A\cdot T_{1}+B)^{2}}$$

(9)

For the final PSD dataset, the quantitative pPSD map was scaled to a value of 5000 inside white matter, i.e. for a pPSD value of approximately 70.

## **Potentiated Proton Spin Density (si^4^_PSD)**

Due to the high signal of various adjacent structures in PSD-weighted images and the potential reduced delineability of the SN resulting from this, a non-linear contrast enhancement was applied. For this purpose, the pPSD values were raised to the power of 4 ($\text{pPSD}^{4}$) to increase the visual contrast:

$$S_{\text{pPSD}^{4}}=(pPSD(T_{1}))^{4}$$

(10)

The noise $\sigma_{\text{pPSD}^{4}}$ for this dataset results from error propagation as follows:

$$\sigma_{\text{pPSD}^{4}}=\left| \frac{\partial S_{\text{pPSD}^{4}}}{\partial T_{1}} \right|\cdot\sigma_{T_{1}}$$

(11)

Derivative with respect to $T_{1}$:

$$\frac{\partial S_{\text{pPSD}^{4}}}{\partial T_{1}}=4\cdot(pPSD(T_{1}))^{3}\cdot\frac{\partial pPSD(T_{1})}{\partial T_{1}}$$

(12)

For the final si^4^_PSD datasets, signal intensities were also scaled to 5000 inside white matter.

## **Inversion-Recovery with WM Nulling (IR_WM)**

As described in Nöth et al.^5^, this synthetic dataset has the signal behavior of an Inversion-Recovery sequence. The calculation was adapted so that the signal from areas with an average white matter (WM) T1 is nulled. The signal intensity $S_{\text{IR}\text{\_}\text{WM}}$ is calculated from the pPSD and qT1 maps:

$$S_{\text{IR\_WM}}=pPSD(T_{1})\cdot\left| 1-2\cdot exp\left( -\frac{ln(2)\cdot T_{1,\text{WM}}}{T_{1}} \right) \right|$$

(13)

To ensure complete regional nulling in the synthetic anatomies, *T*_1,WM_ was determined locally based on the average T1 of the nearest white matter pixels. However, for the noise calculation via error propagation, assuming a fixed mean *T*_1,WM_ of 900 ms is sufficient.

The noise $\sigma_{\begin{aligned} \text{IR\_WM} \end{aligned}}$ is calculated via error propagation:

$$\sigma_{\text{IR\_WM}}=\left| \frac{\partial S_{\text{IR}\text{\_WM}}}{\partial T_{1}} \right|\cdot\sigma_{T_{1}}$$

(14)

Derivative with respect to $T_{1}$ with $A=ln(2)\cdot T_{1,\text{WM}}$:

$$\frac{\partial S_{\text{IR\_WM}}}{\partial T_{1}}=\text{sign}\left( 1-2e^{-\frac{A}{T_{1}}} \right)\cdot\left[ \frac{\partial pPSD(T_{1})}{\partial T_{1}}\cdot\left( 1-2e^{-\frac{A}{T_{1}}} \right)+pPSD(T_{1})\cdot\left( -2e^{-\frac{A}{T_{1}}} \right)\cdot\frac{A}{T_{1}^{2}} \right]$$

(15)

## **Contrast-Increased qT1 Map (ci_T1)**

To assume improved visualization of the Substantia nigra (SN) directly in the underlying qT1 map, a contrast-increased qT1 map was created. For this, the range of typical mean qT1 values ($\bar{T}_{1}$) of the SN was amplified relative to the adjacent WM (AD) using a Fermi filter, leading to a selective increase of up to 100%.

The resulting signal behavior $S_{\text{ci\_T1}}$ is defined as follows:

$$S_{\begin{aligned} \text{ci\_T1} \end{aligned}}=T_{1}\cdot(1+1\cdot F_{\text{Fermi}})$$

(16)

The Fermi filter ($F_{\text{Fermi}}$) was defined as a high-pass filter:

$$F_{\text{Fermi}}=\frac{1}{1+exp\left( -\frac{ln(19)}{\text{extent}}\cdot(T_{1}-\text{cutoff}) \right)}$$

(17)

The parameter $\text{cutoff}$ is defined as $\frac{\bar{T}_{1,\text{SN}}+\bar{T}_{1,\text{AD}}}{2}$ and $\text{extent}$ is defined as $\frac{\bar{T}_{1,\text{SN}}-\bar{T}_{1,\text{AD}}}{2}$.

By design, this high-pass filter yields values ranging from 0 (for short *T*_1_) to 1 (for long *T*_1_), reaching exactly 0.5 at *T*_1_ = cutoff. Furthermore, the filter transitions from 5% to 95% of its maximum value across the interval between *T*_1_ = cutoff – extent and *T*_1_ = cutoff + extent.

A complete analytical derivation of this equation was considered too complex for error propagation.

**MP-RAGE (T1_PSD_MP & T1_MP)**

The derivation of the synthetic MP-RAGE datasets is based on the principles described by Nöth et al.^5^ and is presented here in a simplified manner. The parameters used for the simulation of the synthetic MP-RAGE datasets are listed below.

### (i) Acquisition Parameters

The following acquisition parameters were assumed for the calculation of the synthetic MP-RAGE datasets:

- TR = 1900 ms
- TI: Inversion time = 900 ms
- $\alpha$ = $9^{\circ}$
- es: Echo spacing = 8.1 ms

### (ii) Sequence and Timing Parameters

The calculation of the time intervals required for the signal equation depends on the number of echoes that constitute the acquisition train inside the outer phase encoding loop:

- $n$: Total number of echoes in the train.
- $n_{1}$: Number of the echo inside the train that covers the k-space center.
- $\tau_{1}$: Time interval between the inversion pulse and the start of the readout train with $\tau_{1}=TI-n_{1}\cdot es$
- $\tau_{2}$: Time interval after the readout train with $\tau_{2}=TR-\tau_{1}-(n\cdot es)$

### (iii) Relaxation Parameters

The relaxation factors are calculated from the timing parameters and the T1 time:

- $T_{1}^{*}$: The effective relaxation time during the readout train with $R_{1}^{*}=1/T_{1}^{*}$, calculated as:

$$R_{1}^{*}=R_{1}-\frac{1}{es}ln(cos(\alpha)) \text{with} R_{1}=1/T_{1}$$

(18)

- $q$: The ratio of the effective to the actual relaxation time with $q=T_{1}^{*}/T_{1}$
- $\epsilon_{1}$: Relaxations factor for the interval $\tau_{1}$ with $\epsilon_{1}=e^{-\tau_{1}/T_{1}}$
- $\epsilon_{2}$: Relaxations factor for the interval $\tau_{2}$ with $\epsilon_{2}=e^{-\tau_{2}/T_{1}}$
- $\epsilon_{3}$: Relaxations factor for the entire readout train with $\epsilon_{3}=e^{-(n\cdot es)/T_{1}^{*}}$
- $\epsilon_{4}$: Relaxations factor until k-space center with $\epsilon_{4}=e^{-(n_{1}\cdot es)/T_{1}^{*}}$

### (iv) Signal Calculation

The steady-state magnetization ($M_{ss}$) immediately before the inversion pulse is given by:

$$M_{ss}=\frac{1-\epsilon_{2}+\epsilon_{2}\cdot q-\epsilon_{2}\cdot\epsilon_{3}\cdot q+\epsilon_{2}\cdot\epsilon_{3}-\epsilon_{1}\cdot\epsilon_{2}\cdot\epsilon_{3}}{1+\epsilon_{1}\cdot\epsilon_{2}\cdot\epsilon_{3}}$$

(19)

This yields the magnetization ($M_{i}$) at the time of sampling the k-space center:

$$M_{i}=q\cdot(1-\epsilon_{4})+\epsilon_{4}\cdot(1-\epsilon_{1})-\epsilon_{1}\cdot\epsilon_{4}\cdot M_{ss}$$

(20)

The dataset with PSD influence (T1_PSD_MP) and the purely T1-weighted dataset (T1_MP) are thus calculated as:

$$\begin{aligned} S_{\text{T1\_PSD\_MP}}&=|pPSD(T_{1})\cdot M_{i}| \\ S_{\text{T1\_MP}}&=|M_{i}| \end{aligned}$$

(21)

The two-stage calculation of the MP-RAGE signal equation via $M_{i}$ and $M_{ss}$ corresponds to an algebraic transformation of a signal term $Q$ as given in Eq. (3b) in Nöth et al.^5^. Given the high complexity of the complete MP-RAGE signal equation, an analytical error propagation was omitted.

# **Image Quality**

## **Optical Contrast (OC)**

The Optical Contrast (OC) describes the relative signal difference between two regions, normalized to their mean signal. It is a measure of the visible contrast strength, independent of the absolute signal level or its noise, and correlates with subjective perception^5^.

$$\text{OC}=\frac{|\bar{S}_{A}-\bar{S}_{B}|}{\frac{1}{2}(\bar{S}_{A}+\bar{S}_{B})}$$

(22)

Here, $\bar{S}_{A}$ and $\bar{S}_{B}$ denote the mean signal intensity in region A and B, respectively.

## **Contrast-to-Noise Ratio (CNR)**

The Contrast-to-Noise Ratio (CNR) quantifies the contrast between two regions A and B in relation to the noise. According to Nöth et al.^5^, it is defined as the absolute signal difference divided by the pooled noise $\sigma_{\text{pooled}}=\sqrt{\frac{\sigma_{A}^{2}+\sigma_{B}^{2}}{2}}$:

$$\text{CNR}=\frac{|\bar{S}_{A}-\bar{S}_{B}|}{\sigma_{\text{pooled}}}$$

(23)

Where:

- $\sigma_{A},\sigma_{B}$: The standard deviations of the signal (noise) in regions A and B.

An equivalent formulation results from error propagation considering the standard deviation of the difference signal ($\sigma_{\text{diff}}=\sqrt{\sigma_{A}^{2}+\sigma_{B}^{2}}$):

$$\text{CNR}=\sqrt{2}\cdot\frac{|\bar{S}_{A}-\bar{S}_{B}|}{\sigma_{\text{diff}}}$$

(24)

**Signal-to-Noise Ratio (SNR)**

The Signal-to-Noise Ratio (SNR) is the fundamental measure of image quality within a single, homogeneous region. It is defined as the ratio of the local useful signal (mean intensity) to the local interference signal (noise)^5^.

$$\text{SNR}=\frac{\bar{S}}{\sigma}$$

(25)

Here, $\bar{S}$ denotes the mean signal intensity and $\sigma$ the standard deviation of the signal in the examined region.

**References**

1. Deichmann R. Fast high-resolution T1 mapping of the human brain. Magnetic Resonance in Medicine. 2005;54(1):20–7. https://doi.org/[10.1002/mrm.20552](https://doi.org/10.1002/mrm.20552)
2. Volz S, Nöth U, Jurcoane A, Ziemann U, Hattingen E, Deichmann R. Quantitative proton density mapping: Correcting the receiver sensitivity bias via pseudo proton densities. NeuroImage. 2012;63(1):540–52. https://doi.org/[10.1016/j.neuroimage.2012.06.076](https://doi.org/10.1016/j.neuroimage.2012.06.076)
3. Preibisch C, Deichmann R. T1 mapping using spoiled FLASH-EPI hybrid sequences and varying flip angles. Magnetic Resonance in Medicine. 2009;62(1):240–6. https://doi.org/[10.1002/mrm.21969](https://doi.org/10.1002/mrm.21969)
4. Baudrexel S, Nöth U, Schure JR, Deichmann R. T1 mapping with the variable flip angle technique: A simple correction for insufficient spoiling of transverse magnetization. Magnetic Resonance in Medicine. 2018;79(6):3082–92. https://doi.org/[10.1002/mrm.26979](https://doi.org/10.1002/mrm.26979)
5. Nöth U, Hattingen E, Bähr O, Tichy J, Deichmann R. Improved visibility of brain tumors in synthetic MP-RAGE anatomies with pure T1 weighting. NMR Biomed. 2015;28(7):818–30. https://doi.org/[10.1002/nbm.3324](https://doi.org/10.1002/nbm.3324)
6. Fullerton, G. D., Potter, J. L., & Dornbluth, N. C. (1982). NMR relaxation of protons in tissues and other macromolecular water solutions. Magnetic resonance imaging, 1(4), 209–226. <https://doi.org/10.1016/0730-725x(82)90172-2>
